# The Reusable Load Cell with Protection Applied for Online Monitoring of Overhead Transmission Lines Based on Fiber Bragg Grating

**DOI:** 10.3390/s16060922

**Published:** 2016-06-21

**Authors:** Guoming Ma, Naiqiang Mao, Yabo Li, Jun Jiang, Hongyang Zhou, Chengrong Li

**Affiliations:** 1 State Key Laboratory of Alternate Electrical Power System with Renewable Energy Sources, North China Electric Power University, Beijing 102206, China; lcr@ncepu.edu.cn; 2 Beijing Key Laboratory of High Voltage and EMC, North China Electric Power University, Beijing 102206, China; upc_mao2011@163.com (N.M.); ncepulyb@126.com (Y.L.); 13810222756@163.com (J.J.); 18610369700@163.com (H.Z.)

**Keywords:** ice monitoring, overhead transmission line, fiber Bragg grating, load cell, high sensitivity, overload protection, multi- resolution

## Abstract

Heavy ice coating of high–voltage overhead transmission lines may lead to conductor breakage and tower collapse causing the unexpected interrupt of power supply. The optical load cell applied in ice monitoring systems is immune to electromagnetic interference and has no need of a power supply on site. Therefore, it has become a hot research topic in China and other countries. In this paper, to solve the problem of eccentric load in measurement, we adopt the shearing structure with additional grooves to improve the strain distribution and acquire good repeatability. Then, the fiber Bragg grating (FBG) with a permanent weldable package are mounted onto the front/rear groove of the elastic element by spot welding, the direction deviation of FBGs is 90° from each other to achieve temperature compensation without an extra FBG. After that, protection parts are designed to guarantee high sensitivity for a light load condition and industrial safety under a heavy load up to 65 kN. The results of tension experiments indicate that the sensitivity and resolution of the load cell is 0.1285 pm/N and 7.782 N in the conventional measuring range (0–10 kN). Heavy load tension experiments prove that the protection structure works and the sensitivity and resolution are not changed after several high load (65 kN) cycles. In addition, the experiment shows that the resolution of the sensor is 87.79 N in the large load range, allowing the parameter to be used in heavy icing monitoring.

## 1. Introduction

Heavy ice coating of high–voltage transmission lines may lead to conductor breakage and tower collapse causing electrical supply outages [1,2]. In particular, in the 2008 ice disaster in China, icing on overhead transmission lines and facilities caused outages in some cities and counties with a total population of about 30 million people [3]. Over the past decades, numerous theoretical icing models have been developed in order to create reliable tools for predicting the process features of ice [4]. However, the ice models are limited in different topographical and meteorological conditions, so the online monitoring of ice is more credible. For the conventional electrical load cell used in ice monitoring, it is easily disturbed by strong electromagnetic interference, and it needs a power supply (usually a solar panel) on site. As the optical fiber composite ground wire (OPGW) which is already installed on the electric tower, can be used to transmit the optical signal, an optical ice monitoring system based on FBG is proposed [5,6]. Taking advantages of the fiber Bragg grating (FBG), the system is immune to electromagnetic interference, requires no power supply on site, the chemical properties are stable in harsh environments, and has a long lifespan.

The most important part of the system is the FBG load cell. Yuko Ogawa *et al.* presented a FBG load cell for ice monitoring on overhead transmission lines and carried out the tension experiment to acquire the relationship between the load and strain sensor [7]. However, the temperature compensation of FBG sensing was not considered in their paper. To solve the cross-sensitivity of strain and temperature, an unforced FBG in usually added in the monitoring [8,9], but the cost of the sensor is increased and the number of load cells in series is decreased on account of the extra FBG used for monitoring temperature. In recent years, Min Zhang *et al.* developed a FBG sensor to measure the ice load on a model of phase conductor [10]. However, the simulated conductor is not connected with the phase conductor, the ice load on the model is not the same as that on the phase conductor because of the heat generated by the current in the phase conductor. Yanpeng Hao *et al.* carried out the load experiment on a transmission line tower of the ±800 kV DC transmission line in a test base, but the sensitivity of the sensor is 107.3 N/pm [11]. Since the temperature error with the compensation method is usually 10 pm, the load error caused by temperature is 1073 N. The load error is hard to accept for the ice load monitoring, which is usually several kilonewtons. Although the ice monitoring system based on the FBG sensing technique has been developed in recent years, three problems still need to be solved before field application.

(1) The resolution needs to be improved for small ice load measuring. In the initial icing on the phase conductor, the increment of load caused by ice is only several hundred Newtons. Thus, the sensor resolution needs to be improved.
(2) The load error caused by temperature variation should be reduced. Theoretically, the wavelength shifts of two FBGs mounted on one elastic element is the same under temperature variation. However, field experiments indicate that the wavelength shift difference is about 10 pm under temperature variation [8,12]. In addition, the difference may approach 30 pm in special conditions of strong, cold wind or intense sunlight. Since the load error caused by temperature variation depends on product of the load sensitivity (N/pm) and wavelength shift difference, the load error should be reduced by improving load sensitivity.
(3) Load protection needs to be added to avoid failure under heavy ice load. The mechanical strength request of the FBG load cell is the same as the fitting on the tower of high-voltage overhead transmission lines. For example, the failure load of a sample load cell should be larger than 160 kN according the standard of 110 kV fittings [13], and the effective strain range and sensitivity of a commercial FBG strain gauge is ±2000 µε and 1.4 pm/µε. Thus, the resolution of a sensor without protection is not able to be lower than 57.14 N, otherwise the FBG gauge will fail under heavy load. However, according to (1) the resolution needs to be improved for small ice load measurement. Therefore, load protection needs to be added to balance the resolution and safety range. In addition, for the conventional load cell without protection, the plastic deformation of the elastic element begins after a heavy load impulse, and the repeatability of the load cell is hard to maintain.

In order to solve the problems mentioned above, there is a demand for a novel FBG load cell with several advantages, such as high sensitivity in to a small load scale, reliable mechanical strength for detecting the excessive load, and good repeatability in measurement. In this paper, with shearing structure and a bar-hole protection structure, a novel FBG load cell is presented with variable sensitivity in different load ranges (7.782 N/pm in a small load level and 87.79 N/pm in a large load level) and strong mechanical reliability under heavy load (65 kN).

## 2. Principle of the Load Cell

### 2.1. Principle of FBG Sensing

When a broadband light transmits through the FBG, which is as a type of distributed Bragg reflector, the light at a certain wavelength will be reflected selectively but others will go through the Bragg grating. In this paper, the measurement signal of an optical sensor is the reflective wavelength of an FBG. The parameter of the reflective wavelength is dependent on the effective refractive index (*n_eff_*) and grating period (Λ) of the FBG [14]:

(1) $$\lambda_{B}=2n_{eff}\Lambda$$

The reflective wavelength of the FBG responds to temperature and strain sensitively, and the shift in the FBG wavelength (Δ*λ*) relative with a change in applied strain (Δ*ε*) and a change in temperature (Δ*T*) is approximately given by [15]:

(2) $$\Delta \lambda =K_{\varepsilon }\Delta \varepsilon +K_{T}\Delta T$$

where *K_ε_* is the sensitivity coefficient of FBG responding to the strain changes, and $K_{T}$ is the sensitivity coefficient of the FBG responding to the temperature changes. Thus, the problem of cross-sensitivity of FBGs should be solved in load sensing.

### 2.2. Design of the Load Cell

**Figure 1:**
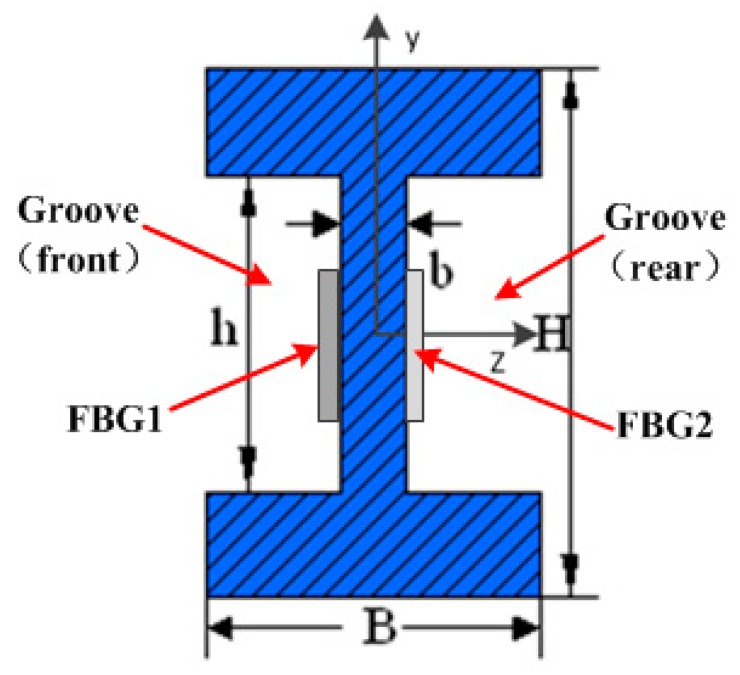
Section view of the elastic element.

The performance of the load cell depends on the structure, and the main units of the proposed load cell are an elastic element and two FBGs as strain sensing components. The material of the elastic element is 35 CrMnSiA. In order to achieve good performance, we adopt the shearing structure coupling with grooves to solve the hysteresis problem of a traditional load cell of the column type. The additional grooves of the new load cell are effective to balance the distribution of strain on the elastic element and increase the accuracy, and the cross-section of the elastic element is an H structure, as shown in Figure 1. The advantages of adopting the H structure are subdued influence of the eccentric load to sensor accuracy and flat strain distribution on the surface of the grooves.

**Figure 2:**
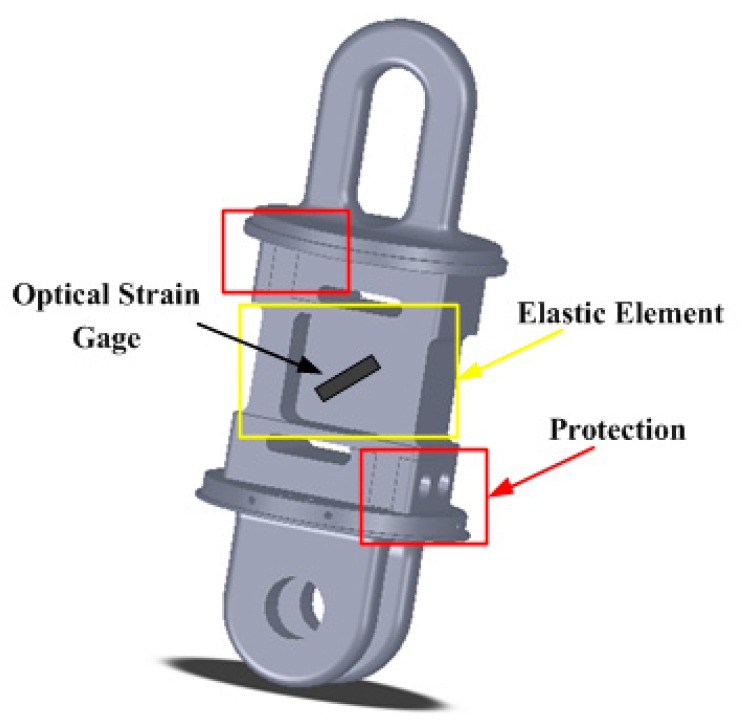
Structure of the load cell.

For linking the electrical towers and insulator strings conveniently, we amended the top and bottom shapes of the elastic elements specifically referring to the traditional fitting of PH-10 and U-10. In addition, for avoiding the breakdown of elastic element, two protecting parts were designed, as seen in the two red rectangles in Figure 2, to achieve the transition of the force structure from an S-shearing structure to a column structure.

### 2.3. Theoretical Calculation of Elastic Element

In theory, the strain on the groove of the elastic element can be calculated under two assumptions:

(a) The direction of shearing stress is in consistent with shearing force; and
(b) The distribution of shearing stress on the cross-section is uniform.

When a force F is applied to the elastic element of the load cell, the shearing stress τ at any point on the H-shape cross area is:

(3) $$\tau =\frac{3F}{2b}\left[\frac{BH^{2}-\left(B-b\right)h^{2}}{Bh^{3}-\left(B-b\right)h^{3}}\right]$$

where H,h,B,b is parameters of H-shape cross section, as seen in Figure 2.

Combining the following formula:

(4) $$\gamma =\frac{\tau }{G}$$

(5) $$G=\frac{E}{2\left(1+\mu \right)}$$

where E is young’s module of elastic element material, G is shear module of elastic element material, and μ is Poisson ratio.

The proximate value of shearing strain in loading experiment is:

(6) $$\gamma =\frac{3\left(1+\mu \right)F}{bE}\left[\frac{BH^{2}-\left(B-b\right)h^{2}}{Bh^{3}-\left(B-b\right)h^{3}}\right]$$

The parameters of the proposed load cell are *E* = 210 GPa, *µ* = 0.3, *H* = 56 mm, *h* *=* 48 mm, *B* = 40 mm, *b* = 14 mm, and the sensitivity in strain of FBG gauge we selected is 1.4 pm/µε. Thus, when the force applied to the load cell is 10 kN (*F* = 10 kN), the resolution of the designed load cell is 8.02 N, according to Equation (6).

### 2.4. FEM (Finite-Element Method) Analysis of the Elastic Element

**Figure 3:**
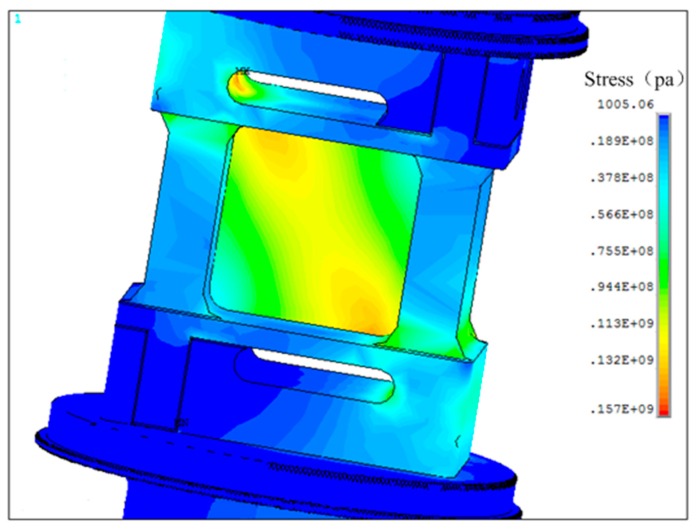
The stress result of elastomer structure.

The finite-element method (FEM) was adopted to observe the direction and value of strain on the surface of the elastic element where the FBGs are welded on. The FEM analysis demonstrated: the maximum shearing stress on the surface of elastic element is 157 Mpa, which is less than the material tension strength of 35 CrMnSiA. Thus, there is no failure in the structure of the load cell that would affect the accuracy and reliability of the sensor; in addition, under the applied axial load of 10 kN in simulation, the gradient field of stress on the key detection unit is along the diagonal direction of the grooves, as shown in Figure 3.

**Figure 4:**
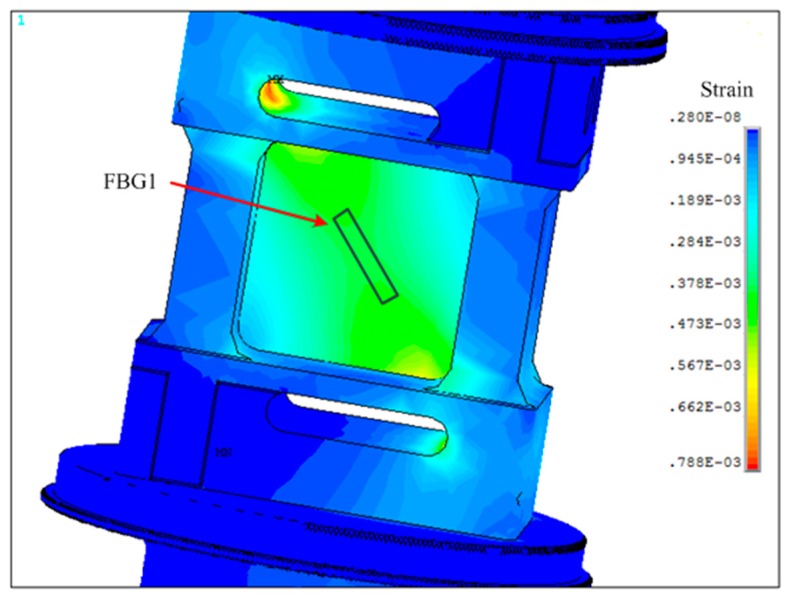
The distribution of stretching strain.

**Figure 5:**
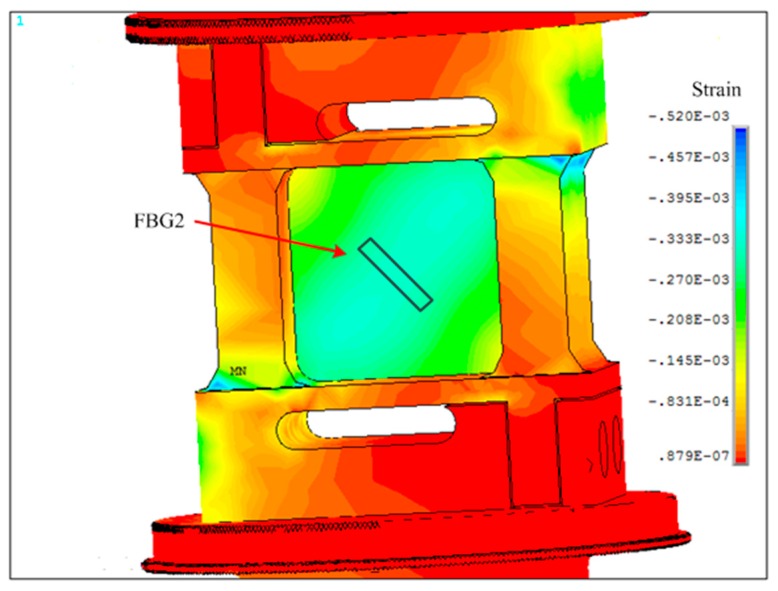
The distribution of compressive strain.

The results of the FEM simulation also indicate the distribution of tension strain and compression strain of the elastic element under the load of 10 kN, seen in Figure 4 and Figure 5. Thus, one FBG was welded at the front groove with the direction deviation of 0° from the gradient field of stress (see in Figure 4), while the other one was welded at the rear groove with the direction deviation of 90° from the gradient field of stress (see in Figure 5). As such, the wavelength shifts of FBGs imposed loading are:

(7) $$\begin{array}{l} \Delta \lambda_{FBG1}=K_{\varepsilon }\Delta \varepsilon_{1}+K_{T}\Delta T\\ \Delta \lambda_{FBG2}=-K_{\varepsilon }\Delta \varepsilon_{2}+K_{T}\Delta T \end{array}$$

The delicate arrangement of the FBGs aims at eliminating the influence of temperature changes by the subtraction between Δ*λ_FBG1_* and Δ*λ_FBG2_*. The FBG wavelength shifts resulting in stresses that are equal in value but opposite in direction. Thus, the wavelength separation shift of two FBGs is:

(8) $$\Delta \lambda =\Delta \lambda_{FBG1}-\Delta \lambda_{FBG2}=K_{\varepsilon }(\Delta \varepsilon_{1}-\Delta \varepsilon_{2})\propto F$$

Here, the wavelength separation shift of two FBGs is proportional to the imposed force while the cross-sensitivity problem of strain and temperature in force sensing is solved. Other temperature insensitive methods can be found in literatures [16,17,18]. Some of them use only one FBG to obtain temperature insensitivity, or use two FBG allows to determine temperature and two direction force. The method we used in the paper depended on the shape of elastic element.

## 3. Temperature Experiment of Load Cell

### 3.1. The Arrangement of Temperature Compensation Experiment

There is a precondition to guarantee the correctness of Equation (8): the sensitivity coefficient of FBGs in temperature (*K_T_*) is the same. However, owing to the processing and installation of FBGs, there is a small difference between two *K_T_* of FBGs mounted on the elastic element. In order to reduce the error resulting from the temperature compensation, we placed the load cell inside a temperature-controlled cabinet to acquire a correction coefficient. The type of the cabinet is GDW-010, made by Shanghai Yishi Instruments and Equipments Factory (Shanghai, China), temperature range is −40 to 150 °C, temperature accuracy is ≤± 0.5 °C, temperature uniformity is ≤± 2 °C.

Under the condition of no imposed force applied to the elastic element, the wavelengths of the FBGs were measured when the temperature was varying from 0 °C to 70 °C then back to 0 °C, and 10 °C as the temperature interval. At each temperature point, there is enough time for thermal transmission of the load cell. The temperature points and reflective wavelength of FBGs were recorded, illustrated in Table 1, and the *λ_1_*, *λ_2_* are the original wavelengths of two FBGs. The Δ*λ_1_*, Δ*λ_2_* are the wavelength shifts of FBGs only inducted by temperature.

**Figure 6:**
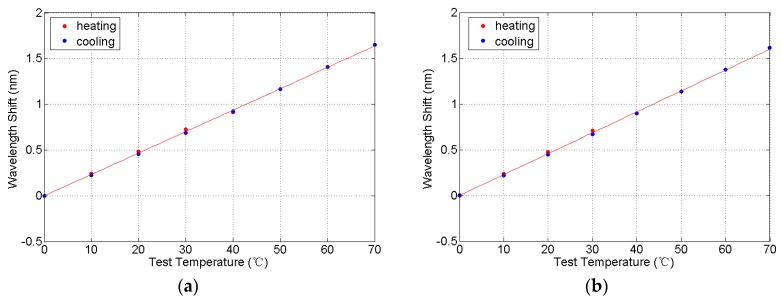
The fitting curve of wavelength shift response to temperature. (**a**) The fitting curve of FBG1 strain; and (**b**) the fitting curve of FBG2 strain.

By fitting the wavelength shifts of FBGs and temperature, we have acquired the equations and the fitting curve which reflect the temperature characteristics of FBGs during heating and cooling in Figure 6:

(9) $$\Delta \lambda_{T1}(nm)=0.02341T({}^{\circ}C)-0.001224$$

(10) $$\Delta \lambda_{T2}(nm)=0.02286T({}^{\circ}C)-0.000993$$

**Figure 7:**
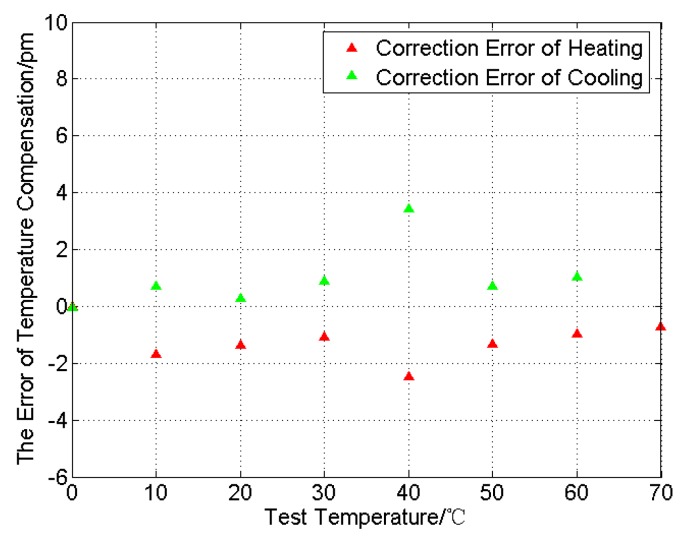
The change in wavelength separations under compensation.

Known from Equations (9) and (10), the slope ratio of two temperature characteristic equations is not consistent, and we proposed a temperature compensation coefficient *k* to guarantee the correctness of Equation (8). When k is 1.024, the wavelength separation (Δ*λ_T_*) in (11) is smaller than ±5 pm, as shown in Figure 7. For the FBGs used for tension sensing, the variation ranges of Δ*λ* is 1280 pm when the applied load is 10 kN; thus, the temperature effect on the load cell performance is less than ±0.39%.

(11) $$\Delta \lambda_{T}=\Delta \lambda_{T1}-k\Delta \lambda_{T2}$$

### 3.2. Outdoor Tempereture Experiment

**Figure 8:**
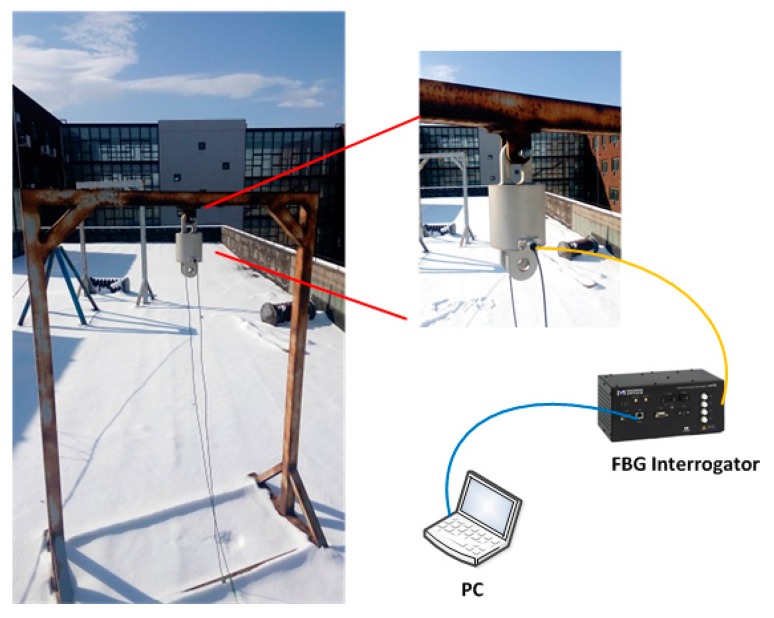
The arrangement of the outdoor experiment.

**Figure 9:**
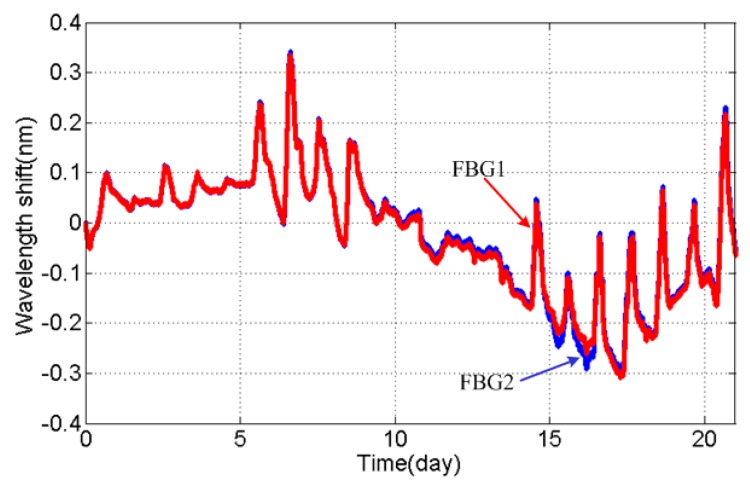
Wavelength shift of FBGs in the outdoor experiment.

Although we have acquired the correction coefficient k, it is necessary to question the reliability of the new load cell in an actual outdoor environment further. Thus, the experiment of placing the load cell on the shelf was carried out and lasted for 21 days, with the setup of the experiment shown in Figure 8. During the outdoor experiment, the monitoring system has recorded the actual wavelength of FBGs mounted on the load cell. Figure 9 shows the original experiment result, and the wavelength shift of FBGs reflects the difference in temperature in day and night well.

**Figure 10:**
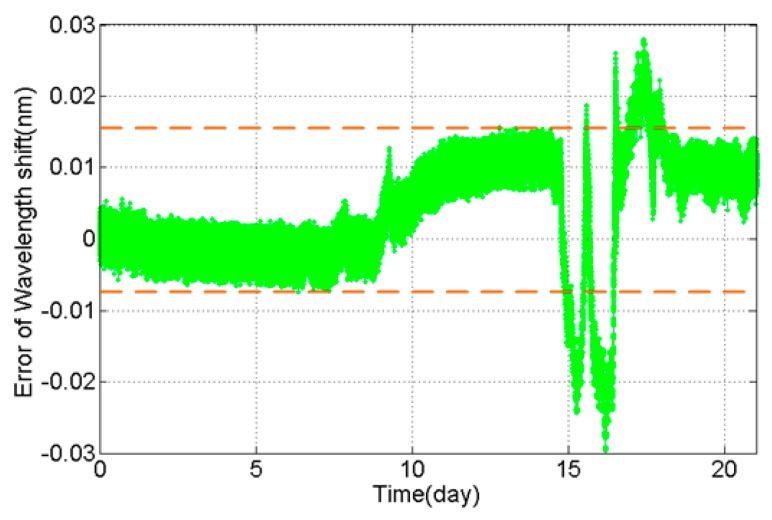
Correction error of FBG wavelength in the outdoor experiment.

As shown in Figure 10, the largest part of correctional wavelength separation is in a small range of −8~15 pm during the outdoor experiment. However, the uneven distribution of the temperature field around the load cell, which was caused by a steady cold wind in the outdoor experiment, would lead to a conspicuous fluctuation extending to 30 pm.

Thus, it is necessary to estimate the measurement accuracy under the fluctuation of 30 pm even if the temperature compensation is effective. For example, for the phase conductor of type LGJ-210/25 (GB 1179-1983), made by Tianjin Tianlan Group Co. Ltd (Tianjin, China), the weight of the conductor with a length of 400 m is around 3156.4 N; the maximum of error resulting in an uneven temperature field is 7.39%, and the error will be decreased in measuring heavy load.

## 4. Tension Experiment of Load Cell

### 4.1. The Arrangement of the Tension Sensing Experiment

**Figure 11:**
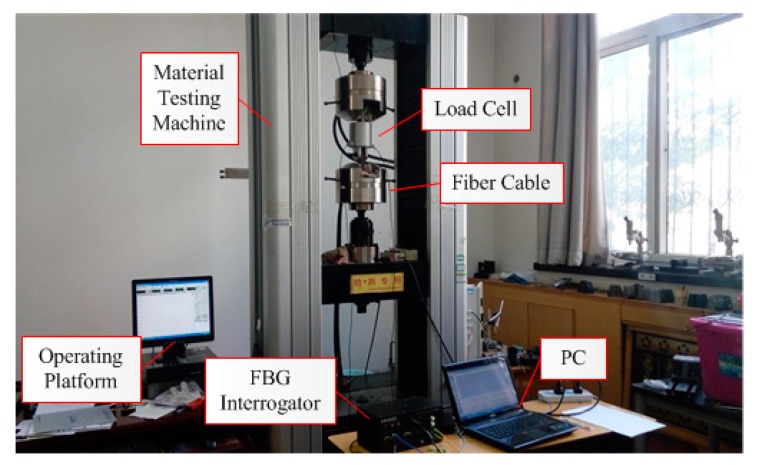
The setup of the tension sensing experiment of a load cell.

The tension sensing experiment was carried out with a high force electromechanical testing machine, which applied the steady force on the sample load cell, as shown in Figure 11. The test procedures referred to standard “JJG 669-2003 verification regulation of Load Cell” [19]. During the tension experiment, the temperature was 20 °C constantly, so the effect of temperature change in the experiment on tension sensing can be reasonably neglected. At the beginning of the tension experiment, we have stretched the load cell repeatedly to remove the residuary stress. Then in the process of the tension sensing experiment, the variable force applied to the load cell was first increased to 10 kN with an increment of 1 kN, then decreased to zero with a decrement of 1 kN.

**Figure 12:**
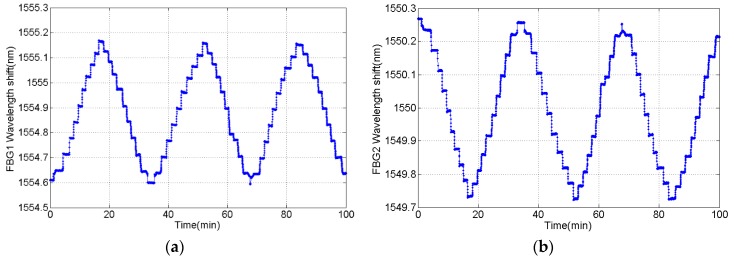
The variation in repeated experiment with alternative load of 10 kN. (**a**) wavelength of stretched FBG1; and (**b**) wavelength of compressed FBG2.

The force was loaded/unloaded three times and the wavelength of two FBGs has been recorded. The tension experiment result indicates that two FBGs wavelength shift are equal in value but opposite in direction, which is correspond with (7), shown in Figure 12. The total wavelength shift (Δ*λ_F_*) was calculated with the equation Δ*λ_F_* = Δ*λ_1_* -*k*Δ*λ_2_* and the processed results of three tension experiments are given in Table 2.

**Figure 13:**
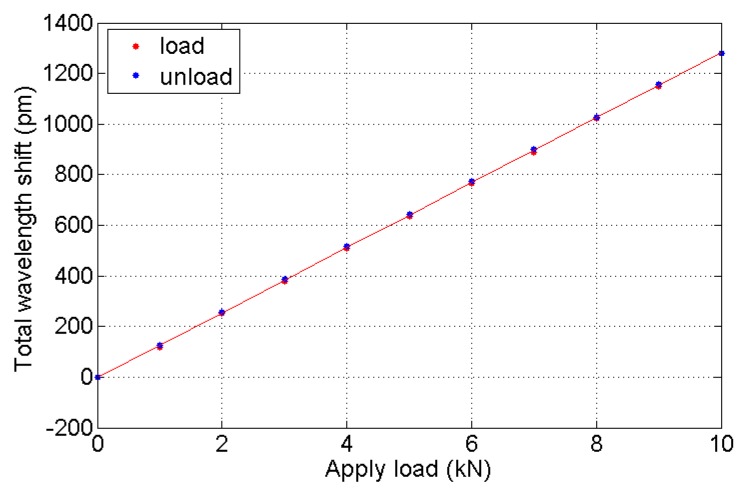
Fitting curve of wavelength shift response to the tension.

As shown in Figure 13, the wavelength shift varies linearly while the applied load changes step by step, and the correlation coefficient is up to 0.9999. Here, the fitting curve which reflects the characteristic relation between wavelength shift and force is:

(12) $$\Delta \lambda_{F1}(pm)=128.52F(kN)-2.84$$

Thus, the sensing sensitivity of the sample load cell is equal to 0.1285 pm/N. The FBG interrogator we used for monitoring the FBG wavelength is sm130, made by Micron Optics Int. (Atlanta, GA USA), and its resolution is 1 pm, so the resolution of the load cell can improve to 7.78 N. In addition, the hysteresis error of the load cell is low, at 0.9337%.

### 4.2. The Overload Examination of Load Cell

The additional protection part in the design of the load cell is necessary to avoid the breakdown of the load cell under heavy load. Tension experiments with heavy load were carried out to confirm the reliability of this load cell. In consideration of the mechanical strength of LGJ-210/25 (65.99 kN in GB 1179-1983) which has been applied widely on 110 kV voltage level overhead transmission lines, the setting maximum of the overload experiment is 65 kN.

**Figure 14:**
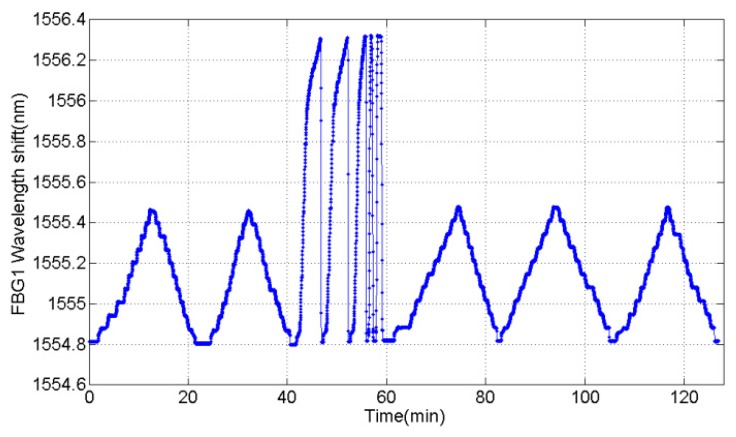
Variation in FBG1 strain in overload experiment.

**Figure 15:**
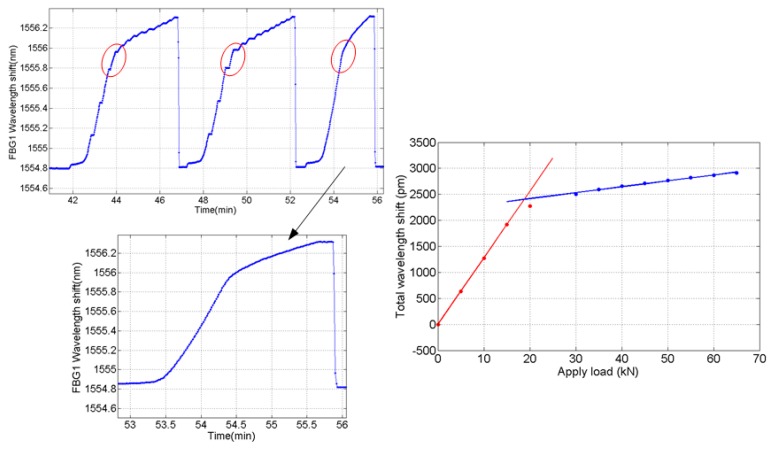
The comparison of reaction of protection architecture.

The wavelength of one FBG during the experiment is illustrated in Figure 14, and the comparison results before and after the experiment clearly indicate that the repeatability of the load cell is great. When the applied load approaches 20 kN, the protection parts act as seen in the three red circle in Figure 15 and the structure of the elastic element turns from the shear type into the column type. There is no obvious damage in the elastic element of the load cell.

After the structure turns into the column type, the load cell can detect the weight of conductor at a large load level with another resolution. The fitting curve which reflects the characteristic of the large load level is (seen in Figure 15):

(13) $$\Delta \lambda_{F2}(pm)=11.39F(kN)+2188$$

Thus, the resolution is calculated by the fitting curve and it is approximately 87.79 N in a large load range.

## 5. Conclusions

(a) A novel structure FBG load cell is proposed for ice monitoring on overhead transmission lines, and the load cell has good performance in ice load measurement, the shear type structure of the elastic element is efficient in transitioning from force into strain. The resolution of the sensor is improved to 7.78 N and hysteresis error of the load cell is reduced to 0.9337%, which makes it possible to detect the thickness of ice in the initial stage of icing.

(b) Temperature compensation is achieved without an extra FBG. Outdoor temperature experiments indicate the maximum error caused by temperature is reduced to 7.39%, and the error will be decreased in measuring heavy load.

(c) With the protection design, the load cell can still work under a large force (up to 65 kN), and it avoids the breakdown between the phase conductor and tower. At a large load level, the resolution of load cell is 87.79 N, and the parameter can be used in heavy icing monitoring. Tension sensing experiments after three 65 kN tension impulses demonstrate the repeatability of the load cell after high load.

## Figures and Tables

**Table 1:** The result of temperature compensation test.

*T* (°C)	*λ_1_* (nm)	*λ_1_* (nm)	Δ*λ_1_* (nm)	Δ*λ_2_* (nm)
0	1554.161	1549.845	0	0
10	1554.402	1550.082	0.241	0.237
20	1554.644	1550.318	0.483	0.473
30	1554.888	1550.556	0.727	0.711
40	1555.075	1550.740	0.914	0.895
50	1555.325	1550.983	1.164	1.138
60	1555.567	1551.219	1.406	1.374
70	1555.812	1551.458	1.651	1.613
60	1555.568	1551.218	1.407	1.373
50	1555.326	1550.982	1.165	1.137
40	1555.085	1550.744	0.924	0.899
30	1554.849	1550.516	0.688	0.671
20	1554.618	1550.291	0.457	0.446
10	1554.387	1550.065	0.226	0.220
0	1554.162	1549.846	0.001	0.001

**Table 2:** Tension experiment result.

F/*kN*	$\Delta \lambda_{F1}$/*pm*	$\Delta \lambda_{F2}$/*pm*	$\Delta \lambda_{F3}$/*pm*
0	0	0	0
1	119	120	118
2	253	253	251
3	382	379	377
4	510	511	509
5	637	636	636
6	764	762	766
7	893	891	890
8	1021	1023	1023
9	1149	1152	1150
10	1281	1279	1281
9	1157	1156	1158
8	1033	1031	1027
7	901	905	902
6	775	774	774
5	649	649	644
4	522	522	518
3	390	391	386
2	262	259	257
1	129	127	125
0	0	0	0
